# Proceedings of the American Philosophical Society, April, Dec., 1850

**Published:** 1851-04

**Authors:** 


*Proceedings of the American Philosophical Society, April, December,* 1850.—Among other interesting matters, we note a letter from Dr. Kane, of the American Arctic Exploring Expedition, now engaged in searching for Sir John Franklin. We are glad to find that there is yet hope for this intrepid traveler and his party.

